# The association of the perioperative fluid balance and cardiopulmonary complications in emergency gastrointestinal surgery: exploration of a randomized trial

**DOI:** 10.1186/s13741-024-00390-y

**Published:** 2024-04-26

**Authors:** Anders W. Voldby, Anne A. Aaen, Ann M. Møller, Birgitte Brandstrup

**Affiliations:** 1 Department of Surgery, Holbæk Hospital, Part of Copenhagen University Hospitals, Smedelundsgade 60, 4300 Holbaek, Denmark; 2 Department of Anesthesiology and Intensive Care Medicine, Holbæk Hospital, Smedelundsgade 60, 4300 Holbaek, Denmark; 3 Department of Anesthesiology and Intensive Care Medicine, Herlev Hospital, Borgmester Ib Juuls Vej 11, 2730 Herlev, Denmark; 4 Department of Clinical Medicine, University of Copenhagen, Blegdamsvej 3B, 2200 Copenhagen N, Denmark

**Keywords:** Fluid therapy, Intestinal obstruction, Intestinal perforation, Intraoperative care, Postoperative complications, Prospective study

## Abstract

**Background:**

The association between perioperative fluid administration and risk of complications following emergency surgery is poorly studied. We tested the association between the perioperative fluid balance and postoperative complications following emergency surgery for gastrointestinal obstruction or perforation.

**Methods:**

We performed a re-assessment of data from the Goal-directed Fluid Therapy in Urgent Gastrointestinal Surgery Trial (GAS-ART) studying intra-operative stroke volume optimization and postoperative zero-balance fluid therapy versus standard fluid therapy. The cohort was divided into three groups at a perioperative fluid balance (FB) of low < 0 L, moderate 0–2 L, or high > 2 L. We used a propensity adjusted logistic regression to analyse the association with cardiopulmonary (primary outcome), renal, infectious, and wound healing complications. Further, the risk of complications was explored on a continuous scale of the FB.

**Results:**

We included 303 patients: 44 patients belonged to the low-FB group, 108 to the moderate-FB group, and 151 to the high-FB group. The median [interquartile range] perioperative FB was –0.9 L [–1.4, –0.6], 0.9 L [0.5, 1.3], and 3.8 L [2.7, 5.3]. The risk of cardiopulmonary complications was significantly higher in the High-FB group 3.4 (1.5–7.6), *p* = 0.002 (odds ratio (95% confidence interval). On a continuous scale of the fluid balance, the risk of cardiopulmonary complications was minimal at –1 L to 1 L.

**Conclusion:**

Following emergency surgery for gastrointestinal obstruction or perforation, a fluid balance < 2.0 L was associated with decreased risk of cardiopulmonary complications without increasing renal complications.

**Supplementary Information:**

The online version contains supplementary material available at 10.1186/s13741-024-00390-y.

## Introduction

Intravenous fluid administration is a central part of perioperative care during emergency gastrointestinal surgery. A too restricted fluid administration may lead to hypovolemia, organ dysfunction, and death (Mythen and Webb 1994; Myles et al. 2018), and a too liberal fluid administration may lead to interstitial oedema, impaired wound healing, and cardiopulmonary complications (Brandstrup et al. 2003; Nisanevich et al. 2005; Abraham-Nordling et al. 2012). As such, the optimal fluid volume follows a U-shaped curve; however, importantly it seems like the optimum varies depending on the specific complications studied (Voldby et al. 2022). A fluid-balance of no more than 2 L is a part of the recommendations of the Enhanced Recovery After Surgery society (ERAS society) during planned abdominal surgery (Ljungqvist et al. 2017). Which fluid balance to aim for during emergency surgery is unknown.

Patients undergoing emergency surgery differ from patients undergoing planned surgery: They tend to be older and have more co-morbidities, and > 40% have sepsis at the time of surgery (Becher et al. 2011). Further, optimizing the fluid balance is challenged by an unknown pre-operative decline in food and fluid intake and pathological fluid losses from vomiting or diarrhoea. Goal-directed fluid therapy (GDT) based on flow related markers possess the theoretic ability to prevent hypovolemic events while avoiding fluid overload. However, studies evaluating the effect of GDT during emergency surgery are few (Harten et al. 2008; Pavlovic et al. 2016; Voldby et al. 2022). A recent retrospective study of patients undergoing emergency gastrointestinal surgery suggested that the fluid balance associated with the lowest number of cardiopulmonary complications was between 0.0 L and 2.0 L (Voldby et al. 2022).

We recently published the results of the randomized Goal-directed Fluid Therapy in Urgent Gastrointestinal Surgery Trial (GAS-ART) testing superiority of GDT over a standard protocol in patients undergoing emergency gastrointestinal surgery. We found no difference in complications and death between the two groups (Voldby et al. 2022). Therefore, we did a re-assessment of the data to evaluate the influence of the perioperative fluid balance on postoperative complications.

The aim of this re-assessment of the GAS-ART data is to test the hypothesis that a peri-operative fluid balance between 0.0 L and 2.0 L is related to the lowest risk of post-operative cardiopulmonary complications following emergency gastrointestinal surgery.

## Methods

This study is an exploration of the results of the GAS-ART trial recently published (Voldby et al. 2022). The GAS-ART trial was approved by the Ethical committee in Region Zealand (SJ-436), and all enrolled patients provided informed consent. The study was categorized as a drug study and registered at EudraCT (no. 2015–000563-14). The rationale and design of the GAS-ART trial were published before study completion (Voldby et al. 2018).

The reporting of these results adheres to the Strengthening the Reporting of Observational Studies in Epidemiology (STROBE) statement.

### Patients

In brief, we included patients between August 2015 and December 2018 undergoing emergency surgery within hours after admittance, for radiologically verified gastrointestinal obstruction or perforation.

The presence of an anaesthesiologist trained in the protocol was mandatory for inclusion. We excluded patients pregnant or younger than 18 years, having a terminal illness (ASA classes 5–6), receiving regular dialysis, with iatrogenic gastrointestinal perforation, unable to give informed consent, or having had intraabdominal surgery within 30 days. Patients were included from five Hospitals located in three regions of Denmark: Odense and Svendborg University Hospitals from Region Southern Denmark, Herlev University Hospital from the Capital Region, and Holbæk and Slagelse Hospitals, both part of Copenhagen University Hospitals from Region Zealand. The randomization was stratified by hospital and by gastrointestinal obstruction or perforation. The number of included patients was based on the power calculation of the original trial. The patients underwent surgery within 6 h of admission; however, in case of ileus, the timing of operation depended on the degree of illness and risk of gangrene and could be postponed until the following day.

### Treatment

A GDT-optimized perioperative fluid regimen was compared with a standard regimen. Preoperative fluid volume and administration was identical between the groups aiming at a heart rate below 100 min^−1^, a systolic blood pressure above 100 mmHg, and venous oxygen saturation above 95%. Intraoperatively, the patients in the GDT-group were given boluses of human albumin 5% in saline based on a stroke-volume algorithm and a maintenance fluid administration ≤ 2 mL kg^−1^ h^−1^; after surgery, the fluid administration aimed at a fluid-balance less than 2 L positive or body weight increase below 2 kg. Patients in the standard-group were intra-operatively given crystalloids to ensure a mean arterial pressure > 65 mmHg and diuresis > 0.5 mL kg^−1^ h^−1^. In both groups, vasopressors were administered to ensure that a mean arterial pressure > 65 mmHg in case the fluid regimen did not achieve that goal. Haemoglobin was kept above 70 g L^−1^, in patients with chronic ischemic heart disease above 80 g L^−1^, or in case of acute ischemic heart disease above 90 g L^−1^.

On the wards, fluid loss was replaced with a fluid that in volume and electrolyte content resembled the fluid lost, aiming at a zero fluid balance in both groups. This was continued until free oral intake, discharge, or the seventh postoperative day in both groups. The GAS-ART trial found a lower intra-operative fluid administration in the GDT-group (1.5L vs. 2.0L) as expected per protocol. The post-operative fluid administration was comparable between the GDT- and the STD-group.

### Explorative analysis

We collected pre-, intra-, and postoperative data prospectively in case report forms.

The fluid input included crystalloids, glucose containing fluids, albumin in saline, packed red blood cells, platelets, fresh frozen plasma, intravenous medicine, and oral fluids. The fluid loss included diuresis, aspirate, vomit, ascites, drain fluid, stoma fluid, perspiration, and blood loss.

In this re-assessment, the exposure variable was the perioperative fluid balance calculated as the difference between the fluid input and loss from induction of anaesthesia and until the end of post-operative day one. We divided the cohort in three groups at a perioperative balance of low < 0L, moderate 0–2L, and high > 2L, because we in a previous study found the 0–2-L interval to be the fluid balance with the lowest complication rate (Voldby et al. 2022).

The primary outcome was cardiopulmonary complications. The secondary outcomes were renal, wound-related, or infectious complications. These outcomes represent our hypotheses for the original trial that the fluid balance influenced cardiopulmonary, wound healing, renal, and infectious complications (Voldby et al. 2018).

The following complications were registered:

- Cardiopulmonary complications: pleural effusion, pulmonary congestion or oedema, respiratory failure requiring mechanical ventilation, arrhythmia, acute myocardial infarction, or cardiac arrest.
- Renal complications: need for renal replacement therapy or other complications needing intervention. In contrast to the original trial, acute kidney injury (AKI) defined according to KDIGO guidelines (increase in plasma creatinine of more than 27 μmol L^−1^ or a 50% increase between a pre-operative creatinine value 30 day prior to surgery and a post-operative value within 48 h) was included in this assessment as a renal complication.
- Wound-related complications: superficial wound rupture or infection, deep wound infection, and fascia defect or dehiscence.
- Infectious complications: superficial or deep wound infection, urinary tract infection, pneumonia, or intraabdominal abscess formation.

The diagnostic criteria of the individual complications are given in the original trial (Voldby et al. 2018; Aaen et al. 2021).

The follow-up for the data used for this assessment was 30 days postoperatively. After discharge, the local investigator contacted the patients by phone if the patient was alive. In addition, the outcomes were registered by blinded assessment of the medical files censored for identity, and all information on fluid therapy, fluid balance, and body weight. Only the assessor blinded outcome data are used in this analysis.

### Statistics

Parametric statistics was used for data following a Gaussian distribution; otherwise, non-parametric statistics was used. Numbers and percentages present categorical variables. The outcome was analysed by qhi (Myles et al. 2018) and logistic regression with the Moderate-FB group as reference.

We used a weighted propensity score for each strata of the comparator. The variables included were chosen by the authors based on a priori knowledge of potential confounders. Continuous variables were age and body-mass-index. Categorical variables were: sex, ASA class (grouped in classes 1–3 or 4–5), excess alcohol intake (> 7 units/week for women and > 14 units/week for men), pre-operative sepsis-2-score (classes 0–2 or 3–4), the surgical method (laparotomy or laparoscopy), the type of surgery (resection of intestine with anastomosis or stoma formation, or no resection of intestine), and the diagnosis (gastrointestinal obstruction, upper perforation (gastric, jejunal or ileac), or lower perforation (colonic or rectal)) and “yes or no” for the following: tobacco use, active cancer, cardiac co-morbidity, pulmonary co-morbidity, other co-morbidity including renal disease, liver disease or diabetes, use of vasopressors, and limited postoperative treatment. A planned adjustment for hospital was abandoned due to low numbers at the three hospitals. Multiple imputation was planned to replace missing values > 5%. We present the crude and adjusted results by odds ratio (OR) with 95% confidence interval (95% CI). Additionally, we present the predicted risk of complications related to the fluid balance on a continuous scale. A generalized additive model with smoothing splines and four degrees of freedom was used. The statistical plan was approved by the authors before commencing the analysing of data. A two-tailed *p* value of less than 0.05 was considered significant. The statistical software was R version 3.5.0 GUI 1.70 El Capitan ©R, 2016 and RStudio version 1.1.453. The codes used can be given on reasonable request.

## Results

A total of 312 patients were randomized in the GAS-ART trial. Three patients withdrew their consent prior to surgery, and five patients did not have surgery. One additional patient partly withdrew consent the day after surgery and is excluded here, but accepted the use of data and follow-up for complications in the original trial, leaving 303 patients for analysis. All patients completed follow-up.

No primary outcome data were missing. Missing values were less than 5% for all covariates, and the propensity score was stable for each comparator. Regarding AKI, creatinine value was missing in four patients (1%) pre-operatively and in five (2%) patients post-operatively. They all had an uneventful post-operative course and are registered event free in the analysis.

The low-FB group included 44 (14.5%) patients, the moderate-FB group 108 (35.6%) patients, and the high-FB group 151 (49.8%) patients. The patients in the low-FB group were slightly younger, but more patients had liver disease or active cancer with a preoperative mutual decision of limited postoperative treatment than patients in the two other groups (Table 1). In the high-FB group, more patients had heart disease, bowel perforation, higher sepsis 2-score, or were transferred directly to the intensive care unit after surgery. The ASA score was comparable between the three groups. Patients receiving GDT were 59% in the low-FB, 57% in the moderate-FB group, and 41% in the high-FB group. During surgery events with a systolic blood pressure < 100 mmHg were more frequent in the low-FB group, while events with a heart rate > 100 min^−1^ were more frequent in the high-FB group (Table 2). Fewer patients were given intra-operative vasopressors in the low-FB group (80%) compared with the moderate- (87%) or high-FB group (87%).

**Table 1:** Background data according to the fluid balance of patients undergoing emergency gastrointestinal surgery

	Low-FB^a^ group, fluid balance < 0.0 L	Moderate-FB group, fluid balance 0.0–2.0 L	High-FB group, fluid balance > 2.0 L
	*n* = 44	*n* = 108	*n* = 151
Sex, female, no (%)	24 (54.5)	55 (50.9)	89 (58.9)
Age, years, median [IQR]	66.0 [56.8, 71.2]	69.0 [57.0, 78.0]	72.0 [61.0, 81.0]
Hospital, no (%)			
Holbaek	5 (11.4)	19 (17.6)	43 (28.5)
Svendborg	0 (0.0)	0 (0.0)	7 (4.6)
Slagelse	0 (0.0)	4 (3.7)	12 (7.9)
Odense	0 (0.0)	9 (8.3)	7 (4.6)
Herlev	39 (88.6)	76 (70.4)	82 (54.3)
Body mass index, median [IQR]	24.8 [20.6, 29.3]	24.3 [22.0, 27.4]	24.1 [21.5, 26.2]
Missing	3	6	17
Actively smoking, no (%)	8 (18.2)	23 (21.3)	40 (26.5)
Excess alcohol intake^b^ no (%)	5 (11.4)	12 (11.1)	18 (11.9)
ASA classification, no (%)			
1–2	26 (59.1)	70 (64.8)	88 (58.3)
3–4	18 (40.9)	38 (35.2)	63 (41.7)
Sepsis 2 score, no (%)			
0–2	43 (97.7)	107 (99.1)	135 (89.4)
3–4	1 (2.3)	1 (0.9)	16 (10.6)
APACHE II score, median [IQR]	14.0 [13.0, 16.0]	14.0 [12.0, 17.0]	15.0 [13.0, 19.0]
Co-morbidity, no (%)			
Heart disease	6 (13.6)	23 (21.3)	42 (27.8)
Hypertension	16 (36.4)	44 (40.7)	57 (37.7)
Pulmonary disease	10 (22.7)	15 (13.9)	28 (18.5)
Renal disease	5 (11.4)	11 (10.2)	13 (8.6)
Liver disease	4 (9.1)	3 (2.8)	2 (1.3)
Diabetes mellitus	4 (9.1)	15 (13.9)	14 (9.3)
Active cancer	10 (22.7)	16 (14.8)	14 (9.3)
Randomization, GDT group, no (%)	26 (59.1)	62 (57.4)	62 (41.1)
Intraabdominal pathology, no (%)			
Ulcer disease	2 (4.5)	7 (6.5)	17 (11.3)
Small bowel perforation	0 (0.0)	3 (2.8)	8 (5.3)
Large bowel perforation	3 (6.8)	9 (8.3)	19 (12.6)
Small bowel obstruction	37 (84.1)	74 (68.5)	85 (56.3)
Large bowel obstruction	2 (4.5)	10 (9.3)	17 (11.3)
Necrosis of intestine	0 (0.0)	2 (1.9)	1 (0.7)
Other^c^	0 (0.0)	3 (2.8)	4 (2.6)
Surgical procedure, no (%)			
Gastro- or duodenoraphia	2 (4.5)	6 (5.6)	17 (11.3)
Adhesiolysis	27 (61.4)	44 (40.7)	49 (32.5)
Resection of small intestine	2 (4.5)	20 (18.5)	29 (19.2)
Resection of large intestine	7 (15.9)	17 (15.7)	28 (18.5)
Other^d^	6 (13.6)	21 (19.4)	28 (18.5)
Resection of intestine or stoma formation, no (%)	17 (38.6)	46 (42.6)	69 (45.7)
Anastomosis			
Small bowel	3 (6.8)	18 (16.7)	30 (19.9)
Ileo-colic	4 (9.1)	12 (11.1)	8 (5.3)
Colo-colic	0 (0.0)	0 (0.0)	2 (1.3)
Laparoscopy, no (%)	7 (15.9)	26 (24.1)	33 (21.9)
Time of anaesthesia, hours, median [IQR]	2.4 [1.8, 3.1]	2.4 [1.6, 3.5]	2.5 [1.9, 3.4]
Time in recovery room, hours, median [IQR]	3.6 [2.7, 6.1]	4.2 [2.8, 6.0]	5.8 [2.8, 12.7]
Missing	2	2	16
Postoperative ICU care, immediately, no (%)	2 (4.5)	5 (4.6)	24 (15.9)
Limited treatment postsurgical, no (%)	3 (6.8)	2 (1.9)	7 (4.6)

^a^Fluid balance. ^b^ > 7 / 14 units week^−1^; women/men. ^c^Intraluminal obstruction of intestine, perforated appendicitis. ^d^Drainage, hernia repair, enterotomy, or stoma formation

**Table 2:** Perioperative fluid administration, losses, and associated variables during emergency gastrointestinal surgery divided according to fluid group

	Low-FB^a^ group (fluid balance < 0.0 L)	Moderate-FB group (fluid balance 0.0–2.0 L)	High-FB group (fluid balance > 2.0 L)
	*n* = 44	*n* = 108	*n* = 151
Intraoperative			
Blood pressure (BP) and heart rate (HR)			
Systolic BP at 1 h, mmHg	100 [92, 113]	108 [95, 120]	103 [92, 120]
Diastolic BP at 1 h, mmHg	52 [49, 58]	55 [48, 61]	54 [47, 60]
HR at 1 h, min^−1^	73 [65, 81]	77 [69, 86]	84 [71, 94]
Systolic BP < 100 mmHg, no (%)	42 (95.5)	99 (91.7)	135 (89.4)
HR > 100 min^−1^, no (%)	6 (13.6)	25 (23.1)	55 (36.4)
Fluid variables, mL, median [IQR]			
Iv^b^ crystalloids	730 [300, 1160]	680 [400, 1010]	1000 [600, 1730]
iv colloids	250 [250, 510]	300 [0, 550]	390 [0, 710]
Other	420 [200, 570]	360 [200, 500]	390 [160, 520]
Total iv fluid administration	1680 [1180, 2160]	1440 [1110, 1960]	2030 [1450, 2700]
Diuresis	260 [100, 500]	150 [40, 270]	180 [60, 300]
Blood loss	0 [0, 200]	0 [0, 100]	0 [0, 50]
Other loss	0 [0, 260]	0 [0, 400]	0 [0, 160]
Total loss	570 [280, 800]	450 [190, 820]	360 [150, 800]
Fluid balance	970 [480, 1430]	910 [640, 1320]	1480 [1000, 2120]
Vasopressor given, no (%)	35 (79.5)	94 (87.0)	132 (87.4)
Ephedrine, no (%)	26 (59.1)	71 (65.7)	85 (56.3)
Dose, mg	25.0 [20.0, 33.8]	20.0 [10.0, 30.0]	20.0 [10.0, 40.0]
Phenylephrine, no (%)	4 (9.1)	60 (55.6)	102 (67.5)
Dose, mg	0.5 [0.2, 0.8]	0.6 [0.4, 1.2]	0.8 [0.4, 1.4]
Norepinephrine, no (%)	4 (9.1)	14 (13.0)	41 (27.2)
Dose, mg	0.6 [0.2, 1.2]	0.2 [0.1, 3.5]	0.3 [0.1, 0.9]
Postoperative			
Fluid variables, mL, median [IQR]			
iv crystalloids	820 [200, 1200]	1010 [500, 2000]	2220 [1100, 3300]
iv colloids	0 [0, 0]	0 [0, 0]	0 [0, 250]
Glucose containing fluids	0 [0, 1000]	0 [0, 1000]	1000 [0, 1680]
Other	1170 [350, 2080]	1330 [570, 2100]	2200 [1420, 3250]
Total iv fluid administration	2460 [1760, 3800]	3140 [2200, 4420]	5430 [4260, 7430]
Diuresis	2250 [1670, 3420]	1720 [910, 2750]	1470 [1030, 2400]
Other loss	1690 [1250, 3560]	1290 [940, 1990]	1340 [960, 2000]
Total loss	5040 [3830, 6170]	3460 [2110, 4420]	3100 [2260, 4340]
Fluid balance	-1960 [-2450, -1540]	80 [-550, 480]	2170 [1320, 3410]
Vasopressor given, no (%)	2 (4.5)	4 (3.7)	14 (9.3)
Perioperative			
Total fluid administration	4380 [3250, 5540]	4880 [3500, 6230]	7820 [6120, 9800]
Total fluid loss	5700 [4110, 7690]	4000 [2480, 5170]	3640 [2620, 5080]
Fluid balance	-870 [-1440, -550]	930 [540, 1330]	3760 [2730, 5290]

^a^Fluid balance. ^b^Intravenous

The median [IQR] perioperative fluid balance was –0.9 L [–1.4, –0.6] in the low-FB group, 0.9 L [0.5, 1.3] in the moderate-FB group, and 3.8 L [2.7, 5.3] in the high-FB group. The post-operative fluid balance was greater in the High-FB group due to a greater administration of crystalloids and glucose-containing fluids combined with less diuresis. The post-operative negative fluid balance in the Low-FB group was mainly due to greater diuresis.

### Primary outcome

The number of patients with a cardiopulmonary complication was 49 (16.2%), of which 4 (9%) were in the low-FB group, 9 (8%) in the moderate-FB group, and 36 (24%) in the high-FB group (*p* = 0,001), primarily due to varying risk of pleural exudation, pulmonary congestion, or respiratory failure (Table 3).

**Table 3:** Risk of complications associated with the perioperative fluid group following emergency gastrointestinal surgery

	Low-FB^a^ group (fluid balance < 0.0 L)	Moderate-FB group (fluid balance 0.0–2.0 L)	High-FB group (fluid balance > 2.0 L)	*p* Value
	*n* = 44	*n* = 108	*n* = 151	
Cardiopulmonary complications	4 (9.1)	9 (8.3)	36 (23.8)	0.001
Arrhythmia, atrial	2 (4.5)	4 (3.7)	6 (4.0)	
Arrhythmia, ventricular	1 (2.3)	0 (0.0)	1 (0.7)	
Acute myocardial infarction	0 (0.0)	1 (0.9)	2 (1.3)	
Cardiac arrest	0 (0.0)	0 (0.0)	1 (0.7)	
Pleural exudation	1 (2.3)	0 (0.0)	9 (6.0)	
Pulmonary congestion	0 (0.0)	2 (1.9)	10 (6.6)	
Respiratory failure	0 (0.0)	2 (1.9)	7 (4.6)	
Renal complications	5 (11.4)	11 (10.2)	25 (16.6)	0.303
Acute kidney Injury^b^	5 (11.4)	10 (9.3)	18 (11.9)	
Hydro nephrosis	0 (0.0)	1 (0.9)	2 (1.3)	
Renal failure demanding dialysis	0 (0.0)	0 (0.0)	5 (3.3)	
Infectious complications	10 (22.7)	29 (26.9)	45 (29.8)	0.633
Superficial wound infection	2 (4.5)	4 (3.7)	8 (5.3)	
Deep wound infection	1 (2.3)	3 (2.8)	1 (0.7)	
Urinary tract infection	2 (4.5)	4 (3.7)	14 (9.3)	
Pneumonia	5 (11.4)	14 (13.0)	21 (13.9)	
Intraabdominal abscess	0 (0.0)	4 (3.7)	1 (0.7)	
Wound-related complications	6 (13.6)	19 (17.6)	17 (11.3)	0.347
Superficial wound rupture	3 (6.8)	9 (8.3)	5 (3.3)	
Superficial wound infection	2 (4.5)	2 (1.9)	5 (3.3)	
Deep wound infection	1 (2.3)	1 (0.9)	1 (0.7)	
Fascia rupture	0 (0.0)	7 (6.5)	6 (4.0)	

The results present number of patients with complications. Only the first appearing complication is presented for the sub-variables of the four groups of complications

^a^Fluid balance. ^b^According to the ‘Kidney Disease: Improving Global Outcome’ (KDIGO) criteria deeming an increase of S-creatinine by > 26.5 mmol/L within 48 h post-surgical

In the adjusted regression analysis, the risk of cardiopulmonary complications was significantly increased in the high-FB group (OR 3.4 (1.5–7.6), *p* = 0.002) but not in the in the low-FB group (OR 1.7 (0.5–6.1), *p* = 0.436) (Table 4).

**Table 4:** Logistic regression analysis on the association between the perioperative fluid group and post-operative complications following emergency gastrointestinal surgery

	Low-FB^a^ group (fluid balance < 0.0L)		Moderate-FB group (fluid balance 0.0–2.0L)	High-FB group (fluid balance > 2.0L)	
	OR^b^ (95% CI)	*p* Value		OR (95% CI)	*p* Value
	Crude analysis				
*Primary outcome*					
Cardiopulmonary complications	1.1 (0.3–3.6)	0.880	Ref^c^	3.4 (1.6–7.9)	0.002
*Secondary outcome*					
Renal complications	1.1 (0.3–3.3)	0.830	Ref	1.7 (0.8–3.9)	0.147
Infectious complications	0.8 (0.3–1.8)	0.598	Ref	1.2 (0.7–2.0)	0.605
Wound related complications	0.7 (0.3–1.9)	0.552	Ref	0.6 (0.3–1.2)	0.149
	Adjusted^d^ analysis				
*Primary outcome*					
Cardiopulmonary complications	1.7 (0.5–6.1)	0.436	Ref	3.4 (1.5–7.6)	0.002
*Secondary outcome*					
Renal complications	1.0 (0.5–1.7)	0.855	Ref	1.7 (0.8–3.6)	0.202
Infectious complications	0.8 (0.3–1.9)	0.571	Ref	1.0 (0.6–1.9)	0.852
Wound-related complications	0.7 (0.3–2.2)	0.577	Ref	0.5 (0.3–1.2)	0.109

^a^Fluid balance. ^b^Odds ratio (95% confidence interval). ^c^The moderate-FB group serves as reference in bi-variate analysis. ^d^Adjusted by a weighted propensity score based on age, BMI, sex, ASA-class (classes 1–3 or 4–5), tobacco use (yes or no), alcohol intake > 7 units/week for women and > 14 units/week for men, pre-operative sepsis-2-score (classes 0–2 or 3–4), yes or no for: active cancer, cardiac co-morbidity, pulmonary co-morbidity, other co-morbidity including renal disease, liver disease or diabetes, limited postoperative treatment, and the use of vasopressors. In addition, laparotomy/laparoscopy, resection or no resection of the intestine, the diagnosis (gastrointestinal obstruction), upper perforation (gastric, jejunal or ileac), or lower perforation (colonic or rectal)

**Fig. 1:**
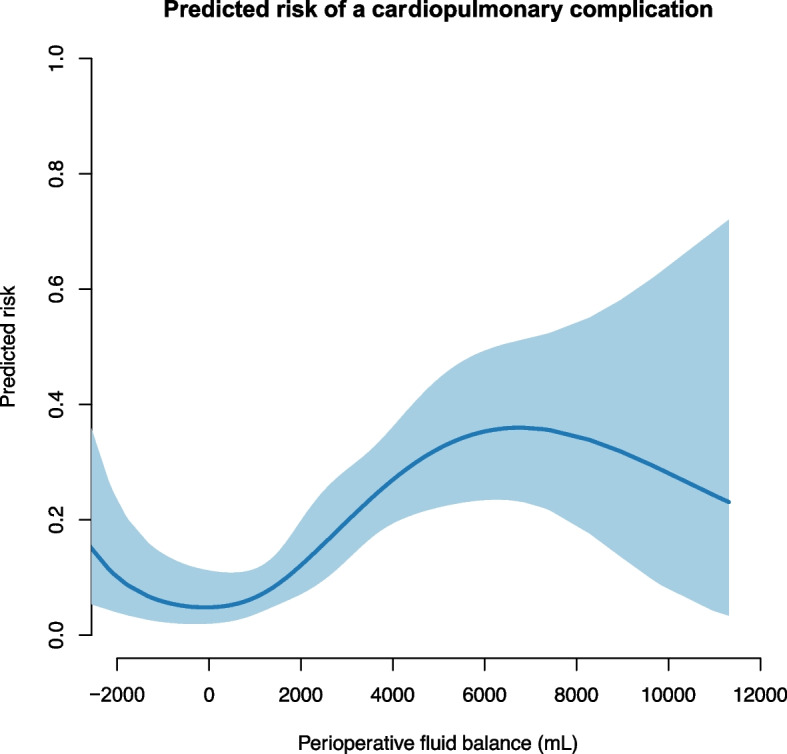
The predicted risk of a cardiopulmonary complication associated with the perioperative fluid balance. The blue line shows the predicted risk of a complication. The shaded area is the 95% confidence interval. We used a generalized additive model with smoothing splines and four degrees of freedom. The parametric effect is *p* < 0.001, and the non-parametric effect is *p* = 0.008. The parametric calculation tests whether the fluid balance is linear associated with complications. The non-parametric analysis tests whether smoothing splines adds further precision to a linear relation of the model

The predicted risk of a cardiopulmonary complication was significantly associated with the perioperative fluid balance on a continuous scale (Fig. 1) and demonstrated a U-shaped relation. A perioperative fluid balance between approximately –1 L and1 L was associated with the lowest risk of cardiopulmonary complications.

### Secondary outcome

**Fig. 2:**
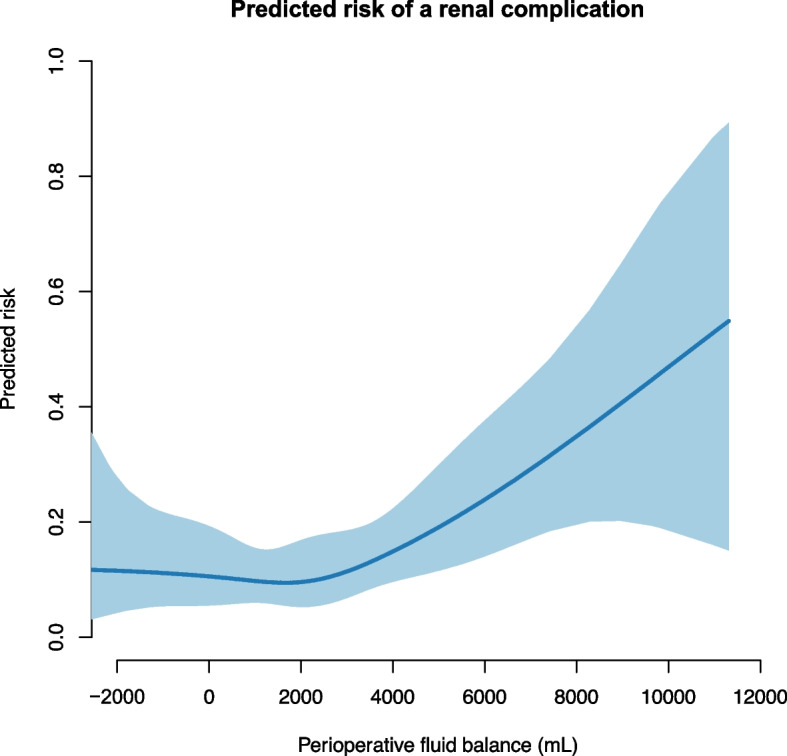
The predicted risk of a renal complication associated with the perioperative fluid balance. The blue line shows the predicted risk of a complication. The shaded area is the 95% confidence interval. We used a generalized additive model with smoothing splines and four degrees of freedom. The parametric effect is *p* = 0.004, and the non-parametric effect is *p* = 0.334. The parametric calculation tests whether the fluid balance is linear associated with complications. The non-parametric analysis tests whether smoothing splines adds further precision to a linear relation of the model

The number of patients with a renal complication were 41 (13.5%) with the greatest number in the high-FB group 25 (16.6%). No significant association was found when comparing the fluid groups. However, the predicted risk of renal complications was significantly associated with the fluid balance on a continuous scale (Fig. 2) and increased at a fluid balance above approximately 3 L.

An overall number of patients with an infectious complication were 84 (27.7%) and with a wound-related complication were 42 (13.9%). We found no significant association between infectious- or wound-related complications and the fluid balance. The spline analysis confirmed this (Supplementary Figs. 1 and 2).

## Discussion

In this secondary analysis of data from a randomized trial of patients undergoing emergency surgery for gastrointestinal obstruction or perforation, we found that the risk of cardiopulmonary complications was significantly associated with a perioperative fluid balance above 2.0 L compared with a fluid balance between 0.0L and 2.0 L. We found that a fluid balance below 0.0 L was not associated with an increased risk of any complications. The findings were solid after propensity score adjustment. The increased risk of cardiopulmonary complications in the high-FB group supports our hypothesis that a perioperative fluid balance above 2 L is associated with an increased risk of complications following emergency gastrointestinal surgery (Voldby et al. 2022).

We found no adverse outcomes from a negative fluid balance of about 1 L. A negative fluid balance may be associated with more hypovolemic events and organ damage. However, 59% of the patients in the low-volume group followed GDT algorithms, which may encompass the ability to prevent hypovolemic events. A pilot study and an early terminated study tested the effect of GDT during emergency surgery and found no increased risk of complications in the group receiving the lowest fluid administration combined with GDT optimisation (Harten et al. 2008; Pavlovic et al. 2016). We found the predicted risk of cardiopulmonary complications to be minimal at a perioperative fluid balance as low as -1 L to 1 L. It is likely that the thorough protocolised postoperative care after surgery in the original trial may have preserved organ function despite an overall perioperative fluid strategy approximating a GDT-zero-balance principle in emergency surgery.

The emergency surgical patient is often in a fluid imbalance on arrival at the hospital. Hence, because of its ability to recognise fluid responsiveness, we believed that the GDT-regimen would be beneficial for the patients. The goal-directed fluid regimen in the GAS-ART trial attempted to prevent hypovolemic events while avoiding fluid overload. Futier and colleagues compared a restrictive GDT-regimen versus a liberal GDT-regimen in elective abdominal surgery. They found more patients with anastomotic leakage, sepsis, or acute lung injury in the restrictive regimen which was argued to be due to more hypovolemic episodes (Futier et al. 2010). In contrast, Lobo and colleagues found significantly fewer complications with fewer cardiovascular and tissue healing events in a restrictive GDT regimen compared to a liberal GDT regimen (Lobo et al. 2011). They argued that a greater administration of colloid boluses in the restrictive group reduced the risk of hypovolemic events and thereby the adverse events. In our study, the administration of albumin was the lowest in the Low-FB group indicating fewer patients with hypovolemic events. Conversely, the administration of albumin was the highest in the high-FB group, as was the number of patients receiving vasopressors, suggesting more hypovolemic events. We found that the risk of cardiopulmonary complications continued to increase with an increase of the fluid balance, which suggests a benefit from a more restrictive fluid approach supporting the findings by Lobo and colleagues.

Of the secondary outcomes, only renal complications were significantly associated with an increase of the fluid balance on a continuous scale. This was surprising to us, because Myles and colleagues found a greater risk of AKI and need for renal-replacement therapy in the group of patients receiving the restrictive fluid regimen compared with a liberal regimen in a trial of elective surgical patients (Myles et al. 2018). Importantly, in the restrictive group, no protocolled action existed for the treatment of oliguria or anuria at the post-operative care unit or at the wards. In comparison, two studies with a well-defined post-operative treatment for oliguria found no increased risk of renal complications from a restrictive fluid regimen (2.6 L to 2.7 L) compared with a liberal regimen (5.0 L to 5.4 L). (Brandstrup et al. 2003; Hübner et al. 2012)

A recent observational study of non-cardiac procedures found that a restrictive as well as a liberal fluid administration was associated with increased risk of renal complications (Shin et al. 2018). An intra-operative fluid administration between 1.8 and 2.7 L had the lowest risk of acute kidney injury. These findings are in agreement with the findings of an observational study of emergency gastrointestinal procedures: a fluid balance between 1.5 and 3.5 L was associated with the lowest risk of renal complication (Voldby et al. 2022). In all, it seems that a positive fluid balance favours renal function, yet a too liberal fluid administration may be harmful as well. Further, our study imply that cardiopulmonary and renal complications is associated unevenly with the fluid balance, which is left for future studies to explore. The strengths of this study are that the data were prospectively collected from multiple centres (increasing generalizability) in a setup with an intra- and postoperative protocolled fluid administration and clearly predefined outcomes. The data collection was monitored by an independent, external “Good Clinical Practice unit”, and the outcome was assessed blinded. Moreover, expecting the data to be screwed towards the sickest patients needing the most IV fluid, we performed a propensity score adjustment of the logistic regression analysis to correct for the most important confounders, including the unevenly distributed risk factors such as sepsis score and bowel perforation.

Our study has limitations. The data originate from a randomized trial, and as such the patients were not treated the same. Some variables differed between the fluid groups, and the result is prone to unknown confounders. Further, it was not possible to adjust for the hospitals in this analysis, due to low numbers at some sites. To our knowledge, no excellent marker exist in clinical practise showing preoperative fluid status accurately. We did not measure blood osmolality for the recognition of dehydration. The fluid balance was based on intra- and post-operative fluid data up to 48 h after surgery only. Fluid administration and loss outside this period may have influenced the outcome. However, the time span for the registered fluid data is in alignment with most other studies in the field. Last but not least, despite correction with a propensity score, the sickest patients with the greatest sepsis score belonged to the high-FB group. It is difficult to separate the influence of this from the influence of the fluid therapy per se. Most of the surgeries in this trial were for small bowel obstruction and a minor part gastrointestinal perforation. In Denmark, perforation caused by colonic tumours have become rare since the introduction of the screening programme for colorectal cancer. The distribution of surgeries may be different in other parts of the world. In conclusion, following emergency surgery for gastrointestinal obstruction or perforation a perioperative fluid balance > 2.0 L was associated with increased risk of cardiopulmonary complications, and the risk was at a minimum at a balance of –1 L to 1 L, without a concomitant increased risk of renal complications. The risk of renal complications was significantly associated with a perioperative fluid balance exceeding 3 L. We found no association between a modest negative fluid balance and complications. No association was found between fluid balance and wound-related or infectious complications.

### Supplementary Information

**Supplementary Material 1.**

**Supplementary Material 2.**

## Data Availability

Following publication of the trials final results, we will make data and materials available after request.
