# Supplementary material for: The association of the perioperative fluid balance and cardiopulmonary complications in emergency gastrointestinal surgery: exploration of a randomized trial
Source: Perioper Med (Lond). 2024 Apr 26;13:32. doi: 10.1186/s13741-024-00390-y (PMC11055263; doi:10.1186/s13741-024-00390-y)
Supplement: Supplementary file 2 — Supplementary Material 2. [file 13741_2024_390_MOESM2_ESM.docx]

Supplementary Figure 2
